# The Effect of AtHKT1;1 or AtSOS1 Mutation on the Expressions of Na^+^ or K^+^ Transporter Genes and Ion Homeostasis in *Arabidopsis thaliana* under Salt Stress

**DOI:** 10.3390/ijms20051085

**Published:** 2019-03-02

**Authors:** Qian Wang, Chao Guan, Pei Wang, Qing Ma, Ai-Ke Bao, Jin-Lin Zhang, Suo-Min Wang

**Affiliations:** 1 Guizhou Institute of Prataculture, Guizhou Academy of Agricultural Sciences, Guiyang 550006, China; snoopy0729@163.com; 2 State Key Laboratory of Grassland Agro-ecosystems, College of Pastoral Agriculture Science and Technology, Lanzhou University, Lanzhou 730020, China; chguan07@lzu.edu.cn (C.G.); maq@lzu.edu.cn (Q.M.); baoaik@lzu.edu.cn (A.-K.B.); jlzhang@lzu.edu.cn (J.-L.Z.); 3 Institution of Qinghai-Tibetan Plateau, Southwest Minzu University, Chengdu 610041, China; wp_10@lzu.edu.cn

**Keywords:** *Arabidopsis thaliana*, *athkt1;1*, *atsos1*, Na^+^, K^+^, salt stress

## Abstract

HKT1 and SOS1 are two key Na^+^ transporters that modulate salt tolerance in plants. Although much is known about the respective functions of HKT1 and SOS1 under salt conditions, few studies have examined the effects of *HKT1* and *SOS1* mutations on the expression of other important Na^+^ and K^+^ transporter genes. This study investigated the physiological parameters and expression profiles of *AtHKT1;1*, *AtSOS1*, *AtHAK5*, *AtAKT1*, *AtSKOR*, *AtNHX1*, and *AtAVP1* in wild-type (WT) and *athkt1;1* and *atsos1* mutants of *Arabidopsis thaliana* under 25 mM NaCl. We found that *AtSOS1* mutation induced a significant decrease in transcripts of *AtHKT1;1* (by 56–62% at 6–24 h), *AtSKOR* (by 36–78% at 6–24 h), and *AtAKT1* (by 31–53% at 6–24 h) in the roots compared with WT. This led to an increase in Na^+^ accumulation in the roots, a decrease in K^+^ uptake and transportation, and finally resulted in suppression of plant growth. *AtHKT1;1* loss induced a 39–76% (6–24 h) decrease and a 27–32% (6–24 h) increase in transcripts of *AtSKOR* and *AtHAK5*, respectively, in the roots compared with WT. At the same time, 25 mM NaCl decreased the net selective transport capacity for K^+^ over Na^+^ by 92% in the *athkt1;1* roots compared with the WT roots. Consequently, Na^+^ was loaded into the xylem and delivered to the shoots, whereas K^+^ transport was restricted. The results indicate that AtHKT1;1 and AtSOS1 not only mediate Na^+^ transport but also control ion uptake and the spatial distribution of Na^+^ and K^+^ by cooperatively regulating the expression levels of relevant Na^+^ and K^+^ transporter genes, ultimately regulating plant growth under salt stress.

## 1. Introduction

Salinity is a major abiotic stress constraining agricultural production worldwide [1,2,3]. A high Na^+^ concentration in the soil principally triggers hyperosmotic stress and disequilibrium in intracellular ion homeostasis, competes with K^+^ uptake in roots, induces secondary stresses, and leads to growth arrest and even plant death [4,5]. However, plants can protect themselves from Na^+^ toxicity through multifarious adaptation strategies, such as restricting Na^+^ entry into roots, excluding Na^+^ from root cells, and sequestering Na^+^ into vacuoles [6,7,8]. Maintaining a high concentration of K^+^ in plants is also an effective strategy to improve salt tolerance under salt stress [9,10]. Therefore, the pivotal mechanism for improving salinity resistance is reducing Na^+^ accumulation and maintaining K^+^ stabilization in plants.

Thus far, two types of transporters have been shown to play vital roles in regulating Na^+^ and K^+^ balance. First, AtHKT1;1 isolated from *Arabidopsis thaliana* has been shown to function as a Na^+^-selective uniporter that controls Na^+^ influx [11]. Knockout of *AtHKT1;1* in *A. thaliana* suppressed the Na^+^ hypersensitivity of the *atsos3* mutant, suggesting that AtHKT1;1 is a salt tolerance determinant that mediates Na^+^ entry [12]. Soil bacterium *Bacillus subtilis* GB03 was found to concurrently down- and upregulate *AtHKT1;1* expression in roots and shoots, respectively, under 100 mM NaCl, resulting in decreased Na^+^ accumulation throughout the plant [13]. Our recent study showed that TEA^+^ and NH_4_^+^ had no significant influence on ^22^Na^+^ influx in wild-type (WT) *A. thaliana* under 25 mM NaCl but reduced ^22^Na^+^ influx by 42% and 46%, respectively, *in athkt1;1*, indicating the key role of AtHKT1;1 in low-affinity Na^+^ uptake [14]. In addition, the known function of AtHKT1;1 is the unloading of Na^+^ from the xylem to xylem parenchyma cells (XPCs). This reduces Na^+^ transport from the roots to the shoots and decreases Na^+^ movement from the xylem into mesophyll cells [15,16,17]. The retrieval of Na^+^ by HKT1 leads to membrane depolarization of XPCs, which activates K^+^ channels, such as SKOR, and indirectly stimulates K^+^ loading into the xylem [18,19,20,21,22]. Therefore, besides acting as a Na^+^ transporter, AtHKT1;1 also regulates K^+^ transport by controlling K^+^ release from the XPCs into the xylem sap toward the shoot indirectly [23,24].

Second, the plasma membrane Na^+^/H^+^ antiporter SOS1 is thought to be involved in Na^+^ efflux in plants [25,26]. SOS1-like efflux activity was found to be higher in salt-tolerant bread wheat (*Triticum aestivum*) than in salt-sensitive wheat, indicating that *TaSOS1* plays a critical role in cytosolic Na^+^ exclusion in wheat [27]. Shi et al. revealed the preferential expression of *AtSOS1* at the plasma membrane of XPCs. AtSOS1 activity was sharply induced by salt stress, which improved Na^+^ efflux to apoplastic spaces and reduced Na^+^ accumulation in the cytoplasm of *A. thaliana* [28]. Na^+^ extrusion from the cytoplasm through AtSOS1 can protect K^+^ uptake mediated by inward rectifying channel AKT1, particularly from impairment by Na^+^ [29]. Furthermore, AtSOS1 may be involved in long-distance transport of Na^+^ from the roots to the shoots, maintaining Ca^2+^ transport and pH homeostasis in plants [28,30]. Similar to AtHKT1;1, AtSOS1 also mediates absorption and transport of other ions indirectly (such as K^+^, Ca^2+^, and H^+^). Thus far, the respective functions of HKT and SOS1 have been mainly identified and characterized at the cell or tissue level; their interactions and roles in regulating Na^+^ and K^+^ homeostasis at the whole-plant level remain unclear. In particular, whether the transcript levels of genes encoding other Na^+^ and K^+^ proteins are affected by mutations of *HKT* or *SOS1* remains unknown.

In the current study, the phenotypes, Na^+^ and K^+^ uptake and accumulation, and the expression patterns of genes encoding important Na^+^ and K^+^ transporters or channels were investigated in WT *A. thaliana* and *athkt1;1* and *atsos1* mutants exposed to salt stress. The results imply that AtHKT1;1 and AtSOS1 not only mediate Na^+^ transport but also control ion uptake and the spatial distribution of Na^+^ and K^+^ by synergistically regulating the expression levels of relevant Na^+^ and K^+^ transporter genes, ultimately regulating plant growth under salt stress.

## 2. Results

### 2.1. Mutation of AtSOS1 and AtHKT1;1 Altered Na^+^ and K^+^ Uptake under Salt Stress

**Figure 1:**
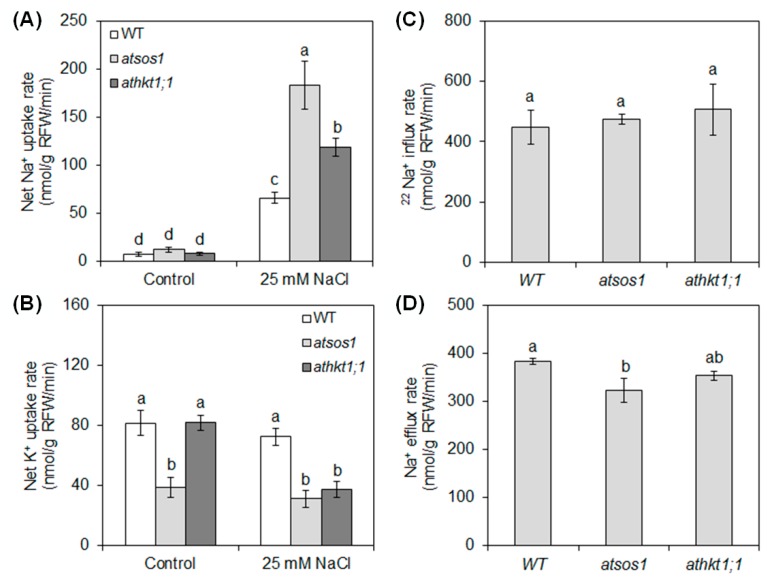
Net Na^+^ (**A**) and K^+^ (**B**) uptake rate in 6-week-old wild type (WT), *atsos1*, and *athkt1;1* with or without 25 mM NaCl for 4 d. ^22^Na^+^ influx rate (**C**) and Na^+^ efflux rate (**D**) in 4-week-old WT, *atsos1*, and *athkt1;1* exposed to 25 mM NaCl. Values are means ±SE (*n* = 8), and bars indicate SE. Columns with different letters indicate significant differences at *p* < 0.05 (Duncan’s test).

We investigated Na^+^ and K^+^ uptake and Na^+^ influx and efflux rate in plant tissues. With increasing NaCl concentration, the net Na^+^ uptake rate increased sharply in WT, *atsos1*, and *athkt1;1* compared with the control (Figure 1A). Interestingly, 25 mM NaCl significantly decreased the net K^+^ uptake rate only in *athkt1;1* compared with the control(Figure 1B). Under control conditions, no significant differences in net Na^+^ uptake rate were found among WT, *atsos1*, and *athkt1;1*, but *atsos1* exhibited a lower net K^+^ uptake rate than the WT (Figure 1A,B). Under 25 mM NaCl, the net Na^+^ uptake rate increased by 177% and 80% in *atsos1* and *athkt1;1*, respectively, compared with WT (Figure 1A). In contrast, the net K^+^ uptake rate decreased by 57% and 49% in *atsos1* and *athkt1;1* under 25 mM NaCl, respectively, compared with WT (Figure 1B). There were no significant differences in the ^22^Na^+^ influx rate among WT, *atsos1*, and *athkt1;1*, but the Na^+^ efflux rate significantly decreased by 16% in *atsos1* compared with WT (Figure 1C,D).

### 2.2. Mutation of AtSOS1 and AtHKT1;1 Altered Na^+^ and K^+^ Partitioning and the Net Selective Absorption and Transport Capacity for K^+^ over Na^+^ in Plant Tissues under Salt Stress

**Figure 2:**
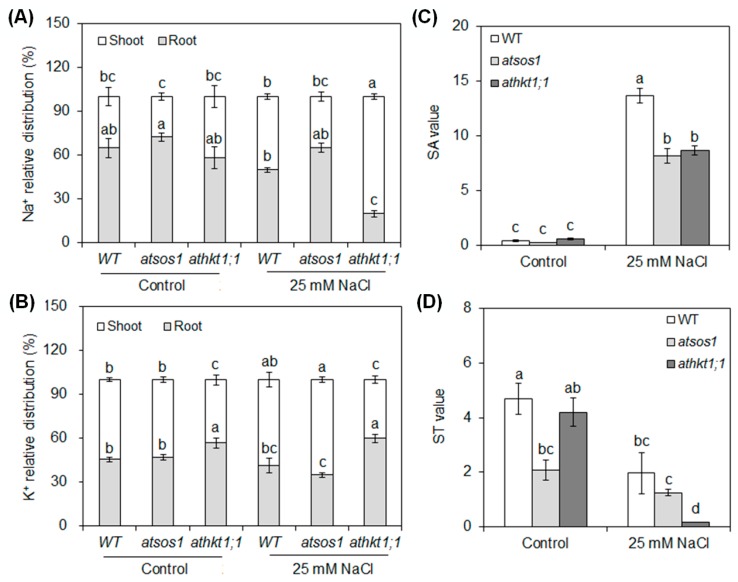
Relative distribution of Na^+^ (**A**) and K^+^ (**B**) in tissues and net selective absorption (**C**) and transport (**D**) capacity for K^+^ over Na^+^ from the roots to the shoots (SA and ST value, respectively) in 6-week-old WT, *atsos1*, and *athkt1;1* with or without 25 mM NaCl for 4 d. Values are means ±SE (*n* = 8), and bars indicate SE. Columns with different letters indicate significant differences at *p* < 0.05 (Duncan’s test).

Compared with the control, an additional 25 mM NaCl decreased the relative distribution of Na^+^ in the *athkt1;1* root and increased it in the *athkt1;1* shoot (Figure 2A). Compared with the relative distributions of K^+^ between control and 25 mM NaCl, the changes were only found in *atsos1* (less in the root and more in the shoot) (Figure 2B). Under control conditions, no significant differences in the relative distribution of Na^+^ were observed among WT, *atsos1*, and *athkt1;1* in the roots and the shoots, but the relative distribution of K^+^ was higher in the *athkt1;1* root (by 25%) and lower in the *athkt1;1* shoot (by 21%) than the WT (Figure 2A,B). An additional 25 mM NaCl decreased the relative distribution of Na^+^ in the *athkt1;1* root and increased it in the *athkt1;1* shoot compared with WT (Figure 2A), while the relative distributions of K^+^ in the *athkt1;1* root and shoot were higher and lower, respectively, than those in WT (Figure 2B).

We further investigated the effects of mutation of *AtSOS1* and *AtHKT1;1* on the net selective absorption capacity for K^+^ over Na^+^ from the roots to the shoots (SA) and the net selective transport capacity for K^+^ over Na^+^ from the roots to the shoots (ST). An additional 25 mM NaCl significantly increased SA in WT, *atsos1*, and *athkt1;1* and decreased ST in WT and *athkt1;1* compared with the control (Figure 2C,D). Under control condition, no significant difference in SA value was found among WT, *atsos1*, and *athkt1;1* (Figure 2C), while *atsos1* exhibited a lower ST value than WT (Figure 2D). The addition of 25 mM NaCl triggered a significant decrease in the SA of both *atsos1* (by 40%) and *athkt1;1* (by 36%) (Figure 2C), decreasing ST by 37% and 92% in *atsos1* and *athkt1;1*, respectively, compared with WT, although the changes were statistically significant only in *athkt1;1* (Figure 2D).

### 2.3. Mutation of AtHKT1;1 and AtSOS1 Altered the Expression Levels of K^+^ and Na^+^ Transporter Genes in Roots under Salt Stress

**Figure 3:**
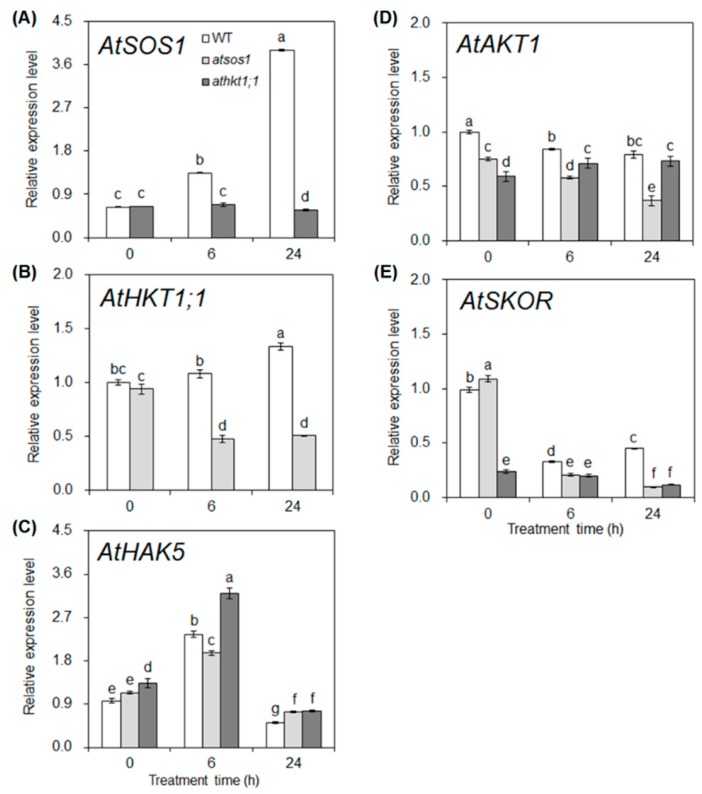
Na^+^ and K^+^ transporter gene expression in WT, *atsos1*, and *athkt1;1* roots exposed to 25 mM NaCl at different times. Quantitative RT-PCR analysis of *AtSOS1* (**A**), *AtHKT1;1* (**B**), *AtHAK5* (**C**), *AtAKT1* (**D**), and *AtSKOR* (**E**) mRNA in roots of 6-week-old WT, *atsos1*, and *athkt1;1* that were treated with 25 mM NaCl for 0, 6, and 24 h. *ACTIN* was used as an internal control. Values are means ±SE (*n* = 9), and bars indicate SE. Columns with different letters indicate significant differences at *p* < 0.05 (Duncan’s test).

For analysis of the expression patterns of K^+^ and Na^+^ transporter genes in the roots under salt stress, plants were subjected to 25 mM NaCl for 0, 6, and 24 h. Under control conditions (0 h), the expression level of *AtSOS1* in *athkt1;1* was the same as that in WT. The additional 25 mM NaCl significantly increased *AtSOS1* expression in WT by 109% and 509% at 6 h and 24 h, respectively, compared with that of plants in the absence of salt. Under salt stress, the expression level of *AtSOS1* in *athkt1;1* was decreased by 49% (6 h) and 85% (24 h) compared with that in WT (Figure 3A). These results suggest that *AtSOS1* is upregulated by salt stress in WT, and the mutation of *AtHKT1;1* triggers a significant downregulation of *AtSOS1* in the roots of *A. thaliana* under saline conditions.

The expression patterns of *AtHKT1;1* in WT and mutant plants with or without salt stress were similar to those of *AtSOS1*. Under control conditions, there was no significant difference in the expression level of *AtHKT1;1* between WT and *atsos1*. Compared with the control (0 h), the addition of 25 mM NaCl increased the expression level of *AtHKT1;1* at 24 h in WT but decreased it by 49% and 46% at 6 and 24 h, respectively, in *atsos1*. Additional NaCl triggered a significant decrease in the expression level of *AtHKT1;1* in *atsos1* compared with WT (Figure 3B). These results indicate that the mutation of *AtSOS1* inhibits *AtHKT1;1* expression in the roots of *A. thaliana* under saline conditions.

Under control conditions (0 h), although there was no significant difference in the expression level of *AtHAK5* between WT and *atsos1*, the expression was 33% higher in *athkt1;1* than in WT. The additional NaCl increased the transcript level of *AtHAK5*, which peaked at 6 h (the highest expression level was observed in *athkt1;1*) and then displayed a decreasing trend in both WT and mutants (Figure 3C). These results suggest that short-term salt stress induces the expression of *AtHAK5*, and the mutation of *AtHKT1;1* triggers a significant upregulation of *AtHAK5* in the roots of *A. thaliana* with or without salt stress.

Under control conditions (0 h), the transcript level of *AtAKT1* was lower and *AtSKOR* was higher in *atsos1* than in WT, while both of these genes were decreased in *athkt1;1* compared with WT (Figure 3D,E). With the increase in treatment time, additional NaCl decreased the expression level of *AtAKT1* in both WT and *atsos1* but increased it in *athkt1;1* (Figure 3D). An additional 25 mM NaCl significantly decreased the transcript level of *AtAKT1* and *AtSKOR* in mutants compared with WT (Figure 3D,E). These results indicate that the expression levels of *AtAKT1* and *AtSKOR* is downregulated in the roots of both *atsos1* and *athkt1;1* under mild salt stress.

### 2.4. Mutation of AtHKT1;1 and AtSOS1 Altered the Expression Levels of K^+^ and Na^+^ Transporter Genes in Shoots under Salt Stress

**Figure 4:**
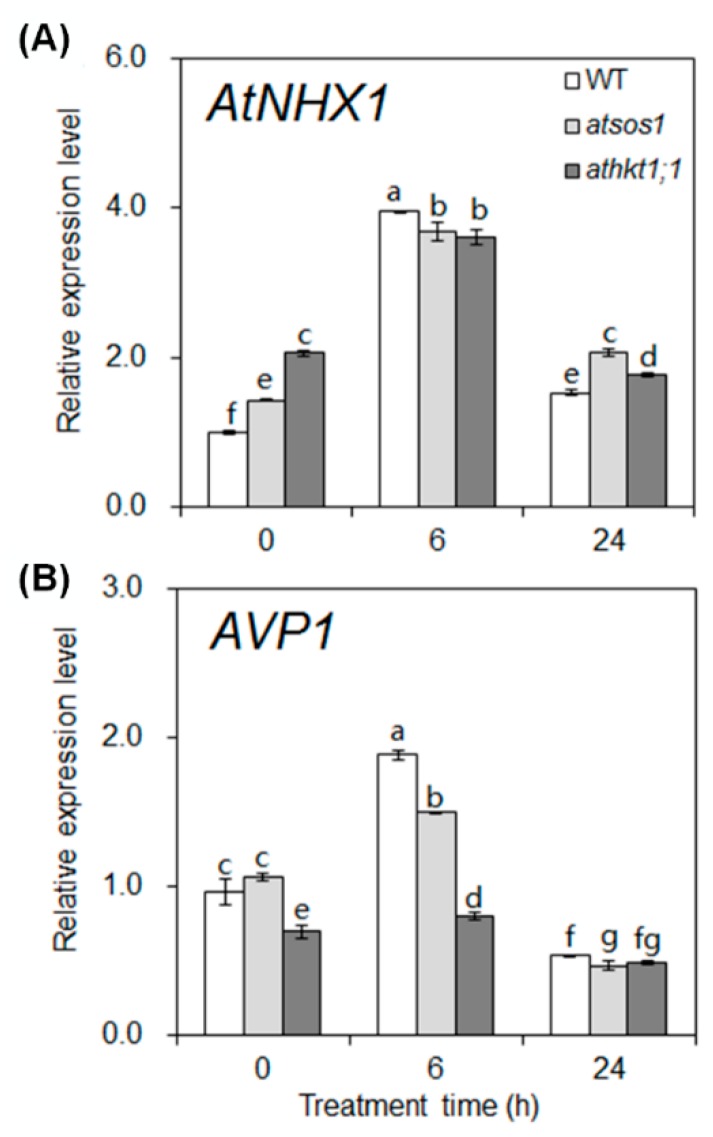
Na^+^ and K^+^ transporter gene expression in WT, *atsos1*, and *athkt1;1* shoots exposed to 25 mM NaCl at different times. Quantitative RT-PCR analysis of *AtNHX1* (**A**) and *AVP1* (**B**) mRNA in shoots of 6-week-old WT, *atsos1*, and *athkt1;1* that were treated with 25 mM NaCl for 0, 6, and 24 h. *ACTIN* was used as an internal control. Values are means ±SE (*n* = 9), and bars indicate SE. Columns with different letters indicate significant differences at *p* < 0.05 (Duncan’s test).

To study the effect of mutation of *AtHKT1;1* and *AtSOS1* on the distribution of K^+^ and Na^+^, we further investigated the expression patterns of K^+^ and Na^+^ transporter genes in the shoots under salt stress. Under control conditions (0 h), the transcript level of *AtNHX1* increased by 43% and 106% in *atsos1* and *athkt1;1*, respectively, compared with WT. With the increase in treatment time, the addition of 25 mM NaCl increased the transcript level of *AtNHX1*, which peaked at 6 h and then displayed a decreasing trend in both WT and mutants (Figure 4A). These results suggest that the mutation of *AtHKT1;1* and *AtSOS1* induces a significant upregulation of *AtNHX1* without salt stress. In addition, the short-term, mild salt stress also triggers an increase in the transcript level of *AtNHX1* in both WT and mutants.

Under control conditions (0 h), there was no significant difference in the expression level of *AtVP1* between WT and *atsos1*, but it was lower by 32% in *athkt1;1* than in WT. With the increase in treatment time, the same change in trend was observed in both WT and mutants. Additional NaCl increased the transcript level of *AtVP1*, which peaked at 6 h and then displayed a decreasing trend. In general, additional NaCl induced an inhibitory effect on the expression of *AtVP1* in both *atsos1* and *athkt1;1* compared with WT (Figure 4B). These results indicate that the mutation of *AtHKT1;1* and *AtSOS1* triggers a significant downregulation of *AtVP1* in the shoots of *A. thaliana* under saline conditions.

## 3. Discussion

### 3.1. Coordination of AtHKT1;1 and AtSOS1 Controls Na^+^ and K^+^ Homeostasis by Regulating Relevant Transporters

**Figure 5:**
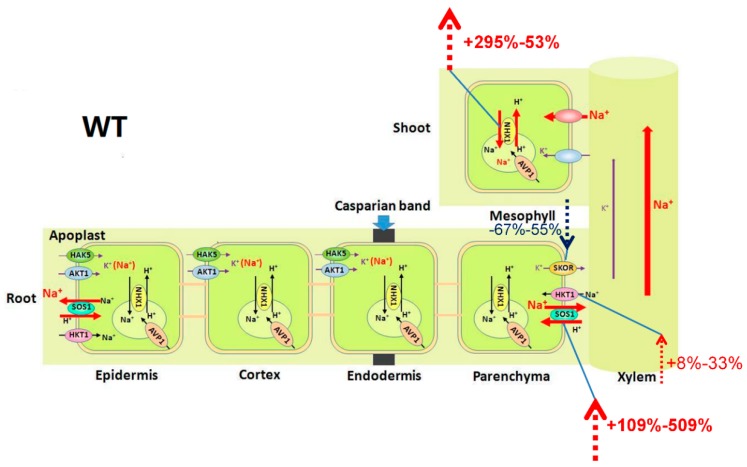
Schematic model for the function of AtHKT1;1 and AtSOS1 in regulating K^+^ and Na^+^ transport in WT of *Arabidopsis thaliana* (*A. thaliana*) under saline conditions. For the plants growing in 25 mM NaCl, the expression level of *AtNHX1* increased 295% at 6 h and 53% at 24 h, indicating that AtNHX1 could efficiently compartmentalize Na^+^ into the vacuoles of the shoots. The addition of 25 mM NaCl induced a 109–509% (6–24 h) increase in transcripts of *AtSOS1* but only an 8–33% (6–24 h) increase in transcripts of *AtHKT1;1* in the roots. At the same time, 25 mM NaCl induced a 67–55% (6–24 h) decrease in transcripts of *AtSKOR* in the roots. Consequently, Na^+^ was loaded into the xylem and delivered to the shoots, whereas K^+^ was restricted.

As a glycophyte, *A. thaliana* is highly sensitive to salinity due to high accumulation of Na^+^ in these plants under salt stress. In our study, an additional 25 mM NaCl enhanced the net Na^+^ uptake rate by 9-fold in WT (Figure 1A) and induced a significant increase in Na^+^ concentration in both the roots and the shoots [31]. However, the plant still grew well under this salt stress (Table S2). At low concentrations (<30 mM NaCl in the case of *A. thaliana*), Na^+^ is beneficial to growth, probably because it serves as a metabolically cheap osmoticum [32]. Vacuoles occupy most of the intracellular volume in many plant cells and are the main cellular reservoir for K^+^. Changes in K^+^ concentration in the tissue are largely a reflection of the dynamics of the vacuolar pool [33]. Na^+^ serves as a cheap osmoticum in the vacuole under salt stress, and changes in concentration of Na^+^ also affects the dynamics of the vacuolar pool. In plants, NHXs catalyzes the electroneutral exchange of Na^+^ and K^+^ for H^+^ using the electrochemical H^+^ gradients generated by the plasma membrane H^+^-ATPases and the vacuolar H^+^-PPase to direct either the movement of Na^+^ or K^+^ out of the cell or the luminal movement of Na^+^ or K^+^ into the vacuoles and intracellular organelles [34]. The NHX gene family of *A. thaliana* includes six members, which are classified into two groups [35,36,37]. One group contains NHX1 to NHX4 and is localized to vacuoles, while the other group contains NHX5 and NHX6 and is localized to the endosomal compartments [38]. In *A. thaliana*, the level of expression of *NHX1* is significantly higher than that of *NHX2* in all the organs examined, and NHX2 expression level is similar to that of other *NHX* members [38]. At the transcript level, *NHX1* and *NHX2* accumulate in response to the same effectors in *A. thaliana* [39,40]. At the protein level, NHX1 and NHX2 are the most closely related members of the NHX family in *A. thaliana*, and they are both localized in the tonoplast [34,39]. Therefore, our study focused on the NHX1 in particular. NHX1 has been shown to mediate Na^+^/H^+^ and K^+^/H^+^ exchange with similar affinity [33,41,42,43], which is assayed under the equimolar concentrations of Na^+^ and K^+^. Under salt stress, an increase in Na^+^ concentration in the tissue may allow NHX1 to compartmentalize cytosolic excess Na^+^ into the vacuole in addition to K^+^ transport because NHX1 lacks significant discrimination between Na^+^ and K^+^ [33,41]. In our work, the expression levels of *AtNHX1* and *AtVP1* swiftly increased by 295% and 71% in WT, respectively, under short-term (6 h) exposure to 25 mM NaCl and then showed a significant decreasing trend (Figure 4A,B). It appears that Na^+^ accumulation in the leaves is probably below the level required for sequestering Na^+^ into the vacuoles under mild salinity [44,45]. The relative distribution of Na^+^ in the shoots with 25 mM NaCl was 42% higher than in conditions without salt stress, although there was no statistically significant change (Figure 2A). The addition of 25 mM NaCl induced a 109% (6 h) to 509% (24 h) increase in transcripts of *AtSOS1* but only an 8% (6 h) to 33% (24 h) increase in transcripts of *AtHKT1;1* in the roots (Figure 3A,B). SOS1 and HKT located at the plasma membrane of XPCs may play crucial roles in regulating Na^+^ transport from the roots to the shoots by mediating opposite fluxes of Na^+^ across the plasma membrane of XPCs [21,46]. Therefore, the ability of AtSOS1 to transport Na^+^ exceeded that of AtHKT1;1, and Na^+^ was loaded into the xylem, which contributed to 42% of the increase in relative distribution of Na^+^ in the shoots (Figure 5).

Although the addition of 25 mM NaCl had no effect on the net K^+^ uptake rate and the relative distribution of K^+^ in the present study (Figure 1B and Figure 2B), our previous work has shown that mild salt stress triggers a significant decrease in K^+^ accumulation in both the shoots and the roots of WT [31]. The transport activity of AtSOS1 outweighs that of AtHKT1;1 at the plasma membrane of XPCs, and Na^+^ is loaded into the transpiration stream under mild salt stress, which would cause membrane hyperpolarization of XPCs and impair K^+^ loading from XPCs into xylem vessels by SKOR, thus decreasing K^+^ transport from the roots to the shoots [20,21,22,23]. In the present work, the addition of 25 mM NaCl induced a 67% (6 h) to 55% (24 h) decrease in *AtSKOR* transcripts in the WT roots (Figure 3E), along with a 58% decrease in ST in WT under mild salt stress (Figure 2D). Therefore, the K^+^ concentration in the shoot decreased by 27% compared with the control (Table S3), which might inhibit K^+^ uptake in a feedback mechanism. AtAKT1 and AtHAK5 have been shown to be major contributors to K^+^ uptake in the roots [47,48]. As the low-affinity pathway, AtAKT1 has the characteristics of channel-mediated transport and dominates K^+^ uptake at external K^+^ concentrations above 0.5 mM [48,49]. Although the SA was induced strongly by mild salt stress in WT (Figure 2B) and the K^+^ concentration in the root had no difference with and without salt stress (Table S3), the addition of 25 mM NaCl decreased the K^+^ concentration in the whole plant by 28% (Table S3) and inhibited *AtAKT1* expression (Figure 3C), which might be attributed to the decrease of K^+^ transport in a feedback mechanism (Figure 5).

Taken together, these data suggest that the coordination of AtHKT1;1 and AtSOS1 regulate the uptake and spatial distribution of Na^+^ and K^+^ and maintain their homeostasis by regulating the expression levels of *AtHKT1;1*, *AtSOS1*, *AtHAK5*, *AtAKT1*, and *AtSKOR* in the roots as well as *AtNHX1* and *AVP1* in the shoots.

### 3.2. AtSOS1 Might Be Essential for Normal Growth and Development of A. thaliana with or without Mild Salt Stress

**Figure 6:**
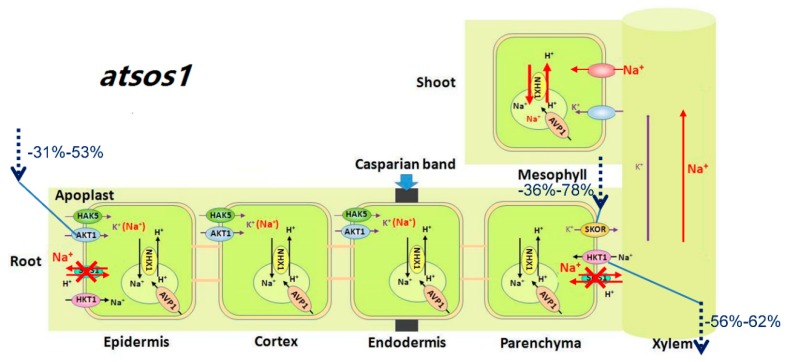
Schematic model for the function of AtHKT1;1 and AtSOS1 in regulating K^+^ and Na^+^ transport in *atsos1* of *A. thaliana* under saline conditions. For the plants growing in 25 mM NaCl, the mutation of *AtSOS1* not only interdicted Na^+^ loading toward shoots but also prevented Na^+^ excretion from the roots to the soil. Compared with WT, loss-of-function of AtSOS1 induced a 56–62% (6–24 h) decrease in transcripts of *AtHKT1;1*, a 36–78% (6–24 h) decrease in transcripts of *AtSKOR*, and a 31–53% (6–24 h) decrease in transcripts of *AtAKT1* in the roots. Consequently, a mass of Na^+^ was accumulated in the roots, whereas K^+^ uptake and transportation was restrained.

Previous research has revealed preferential expression of *AtSOS1* at the plasma membrane of XPCs. The function of AtSOS1 is the loading of Na^+^ directly into the xylem or mediating cytoplasmic Na^+^ efflux to the neighboring apoplastic spaces with reducing Na^+^ accumulation in *A. thaliana* [28]. Under 25 mM NaCl, although there was no difference in the ^22^Na^+^ influx rate between WT and *atsos1* (Figure 2A), the net Na^+^ uptake rate increased by 177% in *atsos1* compared with WT (Figure 1B). These findings indicate that the loss-of-function of AtSOS1 leads to high accumulation of Na^+^ in plants; however, more research is needed to find out which component is responsible for this Na^+^ in plants. Our previous studies have found that there are no differences between WT and *atsos1* in the Na^+^ concentration in the shoot, but Na^+^ accumulation in *atsos1* roots is much higher than that in WT under mild salt stress [31]. The same results were observed in our present study, and the Na^+^ concentration of the whole plant was higher in *atsos1* than in WT (Table S3). At the same time, the relative distribution of Na^+^ with 25 mM NaCl concurrently increased and decreased by 30% in *atsos1* roots and shoots, respectively, compared with that of WT, although there was no statistically significant change (Figure 1C). This finding indicates that the strong Na^+^ increase in *atsos1* roots attributed to Na^+^ transport from the root to the shoot is blocked by the loss of AtSOS1. In addition, the Na^+^ efflux rate was lower in *atsos1* than in WT with 25 mM NaCl (Figure 2B), suggesting that mutation of *AtSOS1* not only interdicted Na^+^ loading toward shoots but also prevented Na^+^ excretion from the roots to the soil under mild salt stress (Figure 6). Therefore, excessive accumulation of Na^+^ in *atsos1* roots would cause growth inhibition, and both the root and shoot biomasses would be seriously impaired compared with those of WT under mild salt stress (Table S2).

Notably, the mutation of *AtSOS1* also inhibited plant growth, even under nonsaline conditions. The fresh weight of the root and the shoot of WT were 25.78 ± 2.96 mg/plant and 202.29 ± 9.60 mg/plant, respectively, in the control, while those of *atsos1* were 15.30 ± 1.17 mg/plant and 159.82 ± 15.21 mg/plant, respectively (Table S2, Figure S1). SOS1 has been suggested to play a critical role in protecting K^+^ uptake mediated by AKT1, on which growth depends. The elevated cytoplasmic Na^+^ level resulting from the loss of SOS1 function impairs K^+^ uptake in root cells and compromises K^+^ nutrition under salt stress [29]. In the present work, *AtAKT1* expression was lower in *atsos1* than in WT with or without salt stress (Figure 3D), accompanied by a decrease in the net K^+^ uptake rate of 53% and 57% with or without salt stress, respectively (Figure 1B). The addition of 25 mM NaCl decreased SA value of *atsos1* by 40% compared with the WT (Figure 2B). Moreover, the ST of *atsos1* was lower than that of WT by 56% and 37% with or without salt stress, respectively (Figure 2D). The decrease of ST in *atsos1* can be attributed to the following two reasons: (1) downregulated expression of *AtAKT1* and *AtHAK5* in *atsos1* (Figure 3C,D), which disturbed K^+^ transport in a feedback mechanism by limiting K^+^ uptake and (2) downregulated expression of *AtHKT1;1* and *AtSKOR* in *atsos1* (Figure 3B,E), which inhibited K^+^ loading directly. Consequently, we conclude that AtSOS1 may play a key role in maintaining normal plant growth with or without salt stress by obtaining the nutrient K^+^ and distributing the toxic Na^+^ (Figure 6).

### 3.3. AtHKT1;1 Might Be the Major Component of Selective Transport Capacity for K^+^ over Na^+^ in A. thaliana under Salt Stress

**Figure 7:**
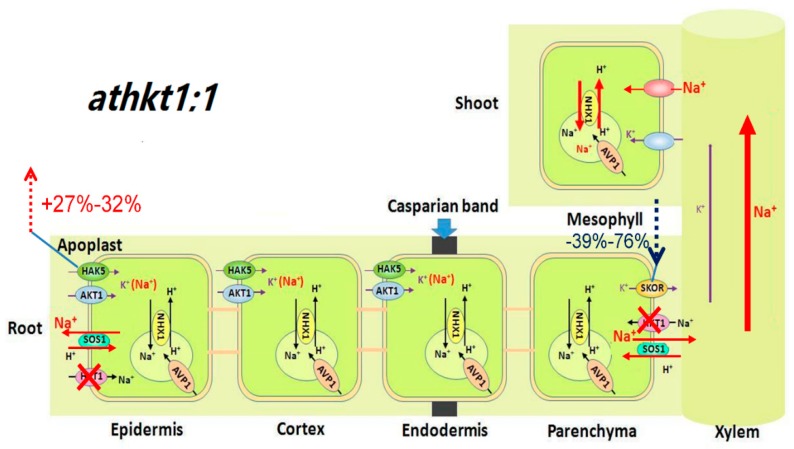
Schematic model for the function of AtHKT1;1 and AtSOS1 in regulating K^+^ and Na^+^ transport in *athkt1;1* of *A. thaliana* under saline conditions. For the plants growing in 25 mM NaCl, mutation of *AtHKT1;1* interdicted Na^+^ unloading from the xylem to the xylem parenchyma cells (XPCs). Compared with WT, loss-of-function of AtHKT1;1 induced a 39–76% (6–24 h) decrease in transcripts of *AtSKOR* in the roots, At the same time, 25 mM NaCl induced a 27–32% (6–24 h) increase in transcripts of *AtHAK5* in the roots, Consequently, Na^+^ was loaded into the xylem and delivered to the shoots, whereas K^+^ was restricted.

The known function of AtHKT1;1 is the unloading of Na^+^ from the xylem to XPCs, which reduces Na^+^ transport from the roots to the shoots and decreases Na^+^ movement from the xylem into the mesophyll cells [15,16,17]. In our study, there was no significant difference in the ^22^Na^+^ influx rate between WT and *athkt1;1* (Figure 2A), but the addition of 25 mM NaCl triggered a strong increase (by 80%) in the net Na^+^ uptake rate in *athkt1;1* compared with WT (Figure 1A). This change might be attributed to the upregulated expression of *AtHAK5* in the roots of *athkt1;1* (Figure 3C). Our previous work showed that AtHAK5 mediated low-affinity Na^+^ uptake in *athkt1;1* [14]. Therefore, the Na^+^ entered into *athkt1;1* roots and was primarily found in *athkt1;1* shoots (Figure 1C and Figure 7), which, due to mutation of *AtHKT1;1*, destroyed the Na^+^ unloading from the xylem to the XPCs and led to accumulation of Na^+^ in the shoots [15,16]. Furthermore, this change caused membrane hyperpolarization of the XPCs and inhibited the activity of the K^+^ channel SKOR, consequently restraining K^+^ loading from XPCs cells into the xylem mediated by SKOR [20,21,22,23]. In the present study, we found that the expression level of *AtSKOR* was downregulated sharply in the roots of *athkt1;1* compared with those of WT under conditions with or without 25 mM NaCl (Figure 3E). Meanwhile, the relative distribution of K^+^ in the shoot was decreased by 21% and 31% with or without salt stress, respectively, in *athkt1;1* compared with WT (Figure 1D). Notably, the ST value was not changed in *athkt1;1* compared with WT under the control condition, while it decreased by 92% compared with the WT under saline conditions. In particular, the addition of 25 mM NaCl dramatically inhibited the ST value in *athkt1;1* (Figure 2D). Recently, Guo et al. found that PtSOS1 is the major component of the selective transport capacity for K^+^ over Na^+^ in *Puccinellia tenuiflora* under salt stress [45]. In our work, the mutation of *AtSOS1* only changed the Na^+^ concentration in different tissues but had no effect on the K^+^ concentration in both the roots and the shoots under salt stress (Table S3) [31]. These findings indicate that, in contrast to PtSOS1, AtSOS1 is a Na^+^ selective transporter. AtHKT1;1 might be the major component for the selective transport capacity for K^+^ over Na^+^ in *A. thaliana* under salt stress.

## 4. Materials and Methods

### 4.1. Plant Materials and Growth Conditions

*Arabidopsis thaliana* materials included EMS-mutagenized *sos1* [28], T-DNA-mutagenized knockout mutant *hkt1;1*, and WT plants [50]. Seeds of WT and *hkt1;1* (*athkt1;1*) and *sos1* (*atsos1*) mutants were surface-sterilized with 75% (*v*/*v*) ethanol and 5% (*v*/*v*) bleach for 3 min and rinsed 5 times with distilled water. Then seeds were vernalized at 4 °C in the dark. Two days later, seeds were planted on solid media containing modified Hoagland nutrient solution (2 mM KNO_3_, 0.5 mM KH_2_PO_4_, 0.5 mM MgSO_4_·7H_2_O, 0.25 mM Ca(NO_3_)_2_·4H_2_O, 1.25 mM CaCl_2_·2H_2_O, 0.06 mM Fe-citrate, 50 μM H_3_BO_3_, 10 μM MnCl_2_·4H_2_O, 1.6 μM ZnSO_4_·7H_2_O, 0.6 μM CuSO_4_·5H_2_O, 0.05 μM Na_2_MoO_4_·2H_2_O) with 1.2% (*w*/*v*) agar and 0.5% (*w*/*v*) sucrose and placed in the greenhouse. Three weeks later, uniform seedlings were transplanted to the black-painted containers with the above Hoagland nutrient solution. The solutions were renewed every 4 days. Seedlings were grown in a greenhouse with a daily photoperiod of 8/16 h (light/dark), a light intensity of 200 μmol m^−2^·s^−1^, a temperature of 22 ± 2 °C/20 ± 2 °C (day/ night), and a relative humidity of 60 ± 5%.

### 4.2. ^22^Na^+^ Influx Experiments

Four-week-old plants were transferred to modified Hoagland nutrient solution supplemented with 25 mM NaCl for 24 h. Then, the seedlings were used to evaluate ^22^Na^+^ influx according to the method described by Essah et al. [51]. Briefly, roots were transferred to the above corresponding solution (10 mL) labeled with 185–370 kBq·L^−1^ of ^22^Na^+^ as the uptake solution. After 2 min, roots were removed from the uptake solution, blotted, and transferred to 200 mL of ice-cold NaCl (25 mM plus 20 mM CaCl_2_) for two successive rinses of 2 min and then another rinse of 3 min. Finally, the roots were blotted gently, weighed, and transferred to glass vials containing 2.5 mL of Optiphase Hisafe (Fisher Chemicals, Loughborough, UK), and the ^22^Na^+^ uptake was determined using a scintillation counter (LS 6000 IC, Reckman Coulter, CA, USA). The ^22^Na^+^ influx was calculated as counts/specific activity/time/root fresh weight (RFW) and expressed as nmol/g RFW/min [52,53].

### 4.3. Calculation of Net Na^+^ and K^+^ Uptake Rate, Na^+^ Efflux Rate, Relative Distribution of Na^+^ and K^+^ in Tissue, and the Net Selective Absorption and Transport Capacity for K^+^ over Na^+^ (SA and ST)

Six-week-old plants were treated with modified Hoagland nutrient solutions supplemented with or without 25 mM NaCl for 4 days. Roots were washed three times for a total of 9 min in deionized water containing 20 mM CaCl_2_ to exchange cell-wall-bound Na^+^, and the shoots were rinsed in deionized water to remove the surface salt [52,54]. The fresh weights of tissues were determined immediately, and the samples were dried in an oven at 70 °C for 3 days to obtain the dry weights of the tissues.

The net uptake rates of Na^+^ and K^+^ were calculated according to the following equation described by Wang et al. [31,52,53]: Using the WT data under control condition as an example, (Na^+^ (or K^+^) content in whole plant of WT under control condition − Na^+^ (or K^+^) content in whole plant of WT before NaCl treatments)/(fresh weight of WT root under control condition × treatment time). The values were expressed as nmol/g RFW/min.

The Na^+^ efflux rate was estimated according to the following formula: Using the control data of WT as an example, (Na^+^ influx of control of WT − Na^+^ net uptake of control of WT)/ (fresh weight of control root of WT × treatment time). The values were expressed as nmol/g RFW/min [53].

The relative distribution of Na^+^ (or K^+^) in tissue was calculated with the following formula: Na^+^ (or K^+^) relative distribution (%) = Na^+^ (or K^+^) content in each tissue/Na^+^ (or K^+^) content in the whole plant [54,55].

The net selective absorption capacity for K^+^ over Na^+^ (SA) from the roots to the shoots was estimated according to the following equation: SA = (K^+^/Na^+^ in whole plant)/(K^+^/Na^+^ in medium) [53,56].

The net selective transport capacity for K^+^ over Na^+^ (ST) from the roots to the shoots was estimated according to the following equation: ST = (K^+^/Na^+^ in shoots)/(K^+^/Na^+^ in roots) [45,54,55,56].

### 4.4. Analysis of the Relevant Gene Expression Level in Roots and Shoots of Wild Type and Mutants

Six-week-old seedlings were treated with modified Hoagland nutrient solution supplemented with an additional 25 mM NaCl, and plants were harvested at 0, 6, and 24 h after treatment. Total RNA was extracted from the roots and the shoots of WT, *athkt1;1*, and *atsos1* using the RNAprep Pure Plant Kit (Tiangen Biotech Co., Ltd, Beijing, China). The purified total RNA was quantified using a Nano-Drop 1000 Spectrophotometer (Thermo scientific, Wilmington, DE, USA). First-stand cDNA was synthesized with MMLV-reverse transcriptase (Takara Biotech Co., Ltd, Dalian, China). The primers were designed using the Primer 5.0 program (Premier Biosoft International, Palo Alto, CA, USA) (Table S1). The *ACTIN* gene was used as an internal control (whose expression was not influenced by salt stress) for data normalization. The expression levels of the *AtSOS1*, *AtHKT1;1*, *AtHAK5*, *AtAKT1*, and *AtSKOR* genes in the roots and the expression levels of *AtNHX1* and *AVP1* genes in the shoots were detected by the ABI PRISM 7500 sequence detection system. SYBR Green PCR master mix (Takara Biotech Co., Ltd, Dalian, China) was used for 20 μL PCR reactions. The condition was 95 °C for 30 s, 40 cycles of 95 °C for 5 s, and 60 °C for 34 s. Each sample was assayed with three independent biological replicates, and each biological replicate was assayed with three technical replicates. The Ct values of target genes and *ACTIN* in different samples were obtained after qRT-PCR reaction. The relative expression levels of genes encoding important transporters or channels normalized to *ACTIN* were calculated using the 2^−ΔΔ*C*t^ method [57].

### 4.5. Statistical Analysis

The ion concentrations and gene expression levels are presented as means with SE and were analyzed by ANOVA using SPSS 16.0 (SPSS Inc., Chicago, IL, USA). Duncan’s multiple range tests were used to detect significant differences between means at a significance level of *p* < 0.05.

## 5. Conclusions

Our studies indicate that AtSOS1 might be essential for normal plant growth and development. AtHKT1;1 might be the major component of selective transport capacity for K^+^ over Na^+^ in *A. thaliana* under salt stress by feedback regulation of the expression of genes involved in Na^+^ and K^+^ transport to mediate ion homeostasis at the whole-plant level. Our results provide a novel idea to systematically investigate the cooperative role of various ion transporters in alleviating Na^+^ toxicity. Further studies at the protein level in double mutants are worthwhile to uncover the detailed mechanism and to search for the coregulators of these transporters.

## Supplementary Materials

Supplementary materials can be found at https://www.mdpi.com/1422-0067/20/5/1085/s1.

## Abbreviations

AKT	Arabidopsis K^+^ transporter
ANOVA	Analysis of variance
AVP	Arabidopsis H^+^-pyrophosphatase
Ct	Threshold cycle
HAK	High-affinity K^+^ transporter
HKT	High-affinity K^+^ transporter
NHX	Tonoplast Na^+^/H^+^ antiporter
PCR	Polymerase chain reaction
SE	Standard error
SKOR	Stelar K^+^ outward rectifier
SOS1	Plasma membrane Na^+^/H^+^ antiporter
WT	Wild type
