# Supplementary material for: The Effect of AtHKT1;1 or AtSOS1 Mutation on the Expressions of Na+ or K+ Transporter Genes and Ion Homeostasis in Arabidopsis thaliana under Salt Stress
Source: Int J Mol Sci. 2019 Mar 2;20(5):1085. doi: 10.3390/ijms20051085 (PMC6429264; doi:10.3390/ijms20051085)
Supplement: Supplementary file 1 [file ijms-20-01085-s001.pdf]

International Journal of *Molecular Sciences*

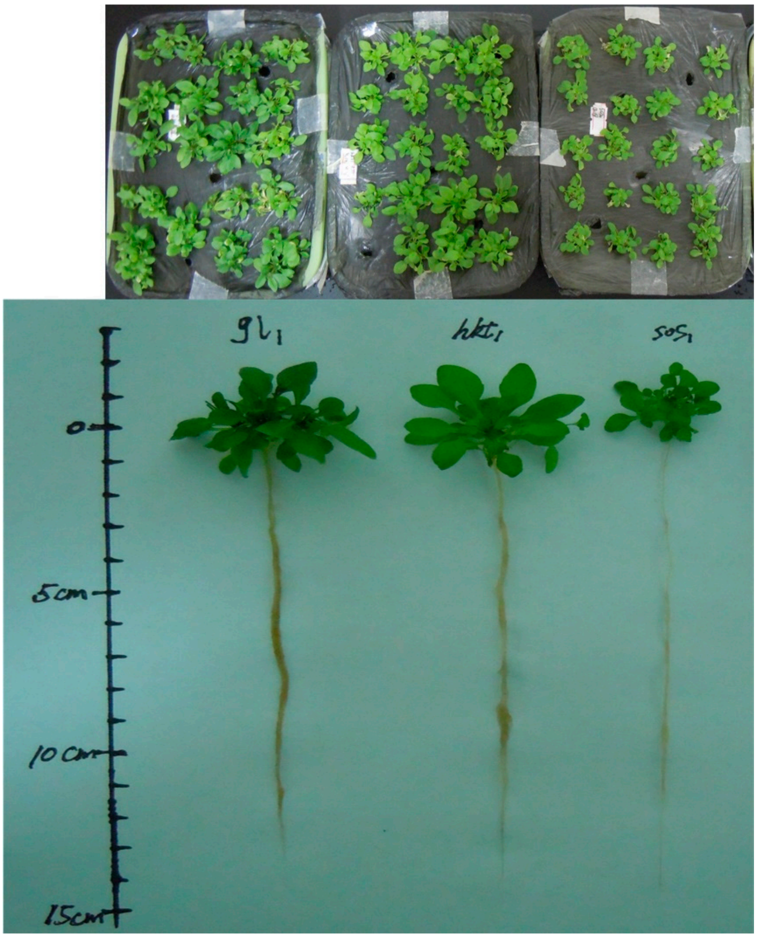

Figure S1. The phenotypic pictures of the plants without salt stress.

Table S1. Sequences of primers used for quantitative real-time PCR amplification and the resulting product size.

Primer	Sequence (5'-3')	Amplicon Length (bp)	Gene
P1	GATTTGTCCCCACGAATGAGA	136	*AtSOS1*
P2	TGCGAAGAAGGCGTAGAACA		
P3	GATTTGTCCCCACGAATGAGA	116	*AtHKT1;1*
P4	CAAAACCAAGAAGCAAGGGAAC		
P5	CCGCCCATTCATCTTCGT	107	*AtHAK5*
P6	CATCCCTTTCTCTCTCGCTTTC		
P7	AAAGGTCTCACTCATCAACAACGA	89	*AtAKT1*
P8	TCGGCAAAAGAGGCAAAATAAG		
P9	ACCGAAACAAACTCGGTAGGAA	121	*AtSKOR*
P10	TTAGCACGGATAGAGACAGGAATG		
P11	ATTGAGCCTTCAGGGAACCA	141	*AtNHX1*
P12	AAAGCCACGACCTCCAAAGA		
P13	GGCTTACCGACCTCCTTTACC	93	*AVP1*
P14	CCAAAGACCAACACAAACACAA		
A1	GCACCGCCAGAGAGAAAATAC	170	*ACTIN*
A2	CACCACCACGAACCAGATAAGA		

Table S2. Fresh weight and dry weight and tissue water content in 6-week-old WT, *atsos1* and *athkt1;1* with or without 25 mM NaCl for 4 days. Values are means ±SE ($n = 8$) and bars indicate SE. Columns with different letters indicate significant differences at $p < 0.05$ (Duncan's test).

Header	Header	Fresh Weight (mg/plant)		Dry Weight (mg/plant)		Tissue Water Content (g/g DW)	
Header	Header	Control	25 mM NaCl	Control	25 mM NaCl	Control	25 mM NaCl
Root	WT	25.78 ± 2.96 b	31.65 ± 2.19 ab	2.00 ± 0.20 ab	2.30 ± 0.09 a	11.76 ± 0.63 b	12.66 ± 0.57 b
	atsos1	15.30 ± 1.17 c	5.21 ± 0.59 d	1.10 ± 0.09 bc	0.56 ± 0.07 c	13.20 ± 0.65 b	8.76 ± 1.10 c
	athkt1;1	36.59 ± 4.03 a	38.42 ± 3.19 a	2.95 ± 0.52 a	2.3 ± 0.15 a	12.67 ± 0.80 b	15.59 ± 0.83 a
Shoot	WT	202.29 ± 9.60 ab	206.98 ± 8.54 a	13.46 ± 1.06 a	15.91 ± 0.78 a	14.77 ± 0.75 a	12.05 ± 0.25 b
	atsos1	159.82 ± 15.21 c	77.74 ± 3.94 d	9.84 ± 0.65 b	6.89 ± 0.31 c	14.78 ± 0.56 a	10.28 ± 0.23 b
	athkt1;1	236.94 ± 9.92 a	167.49 ± 11.10 bc	15.69 ± 0.79 a	13.86 ± 0.79 a	14.36 ± 0.64 a	11.06 ± 0.30 b

Table S3. Na^+ and K^+ concentrations of roots and shoots and the whole plants in 6-week-old WT, *atsos1* and *athkt1;1* with or without 25 mM NaCl for 4 d. Values are means ±SE ($n = 8$) and bars indicate SE. Columns with different letters indicate significant differences at $p < 0.05$ (Duncan's test).

Header	Header	Na^+ Concentration (mmol/g.DW)		K^+ Concentration (mmol/g.DW)	
Header	Header	Control	25 mM NaCl	Control	25 mM NaCl
Root	WT	0.25 ± 0.05 d	0.68 ± 0.02 b	1.22 ± 0.05 b	0.81 ± 0.10 bc
	atsos1	0.34 ± 0.05 cd	1.37 ± 0.16 a	1.25 ± 0.09 b	0.58 ± 0.04 c
	athkt1;1	0.20 ± 0.04 d	0.46 ± 0.06 c	2.04 ± 0.23 a	1.19 ± 0.13 b
Shoot	WT	0.08 ± 0.01 c	0.68 ± 0.04 b	1.45 ± 0.05 a	1.06 ± 0.06 b
	atsos1	0.12 ± 0.02 c	0.72 ± 0.08 b	1.38 ± 0.03 a	1.08 ± 0.02 b
	athkt1;1	0.09 ± 0.01 c	1.80 ± 0.05 a	1.37 ± 0.04 a	0.77 ± 0.03 c
Whole plant	WT	0.10 ± 0.01 d	0.60 ± 0.08 c	1.42 ± 0.04 a	1.02 ± 0.05 b
	atsos1	0.14 ± 0.02 d	0.77 ± 0.07 b	1.36 ± 0.03 a	1.04 ±0.02 b
	athkt1;1	0.11 ± 0.01 d	1.61 ± 0.03 a	1.43 ± 0.15 a	0.83 ± 0.03 c

© 2019 by the authors. Submitted for possible open access publication under the terms and conditions of the Creative Commons Attribution (CC BY) license (http://creativecommons.org/licenses/by/4.0/).
